# Penetrating chest trauma in Sauer's danger zone without serious heart or lung damage: An unusual case report

**DOI:** 10.1016/j.ijscr.2022.106843

**Published:** 2022-02-18

**Authors:** Hideomi Ichinokawa, Yasuhito Konishi, Sinsuke Uchida, Kenji Suzuki

**Affiliations:** Department of General Thoracic Surgery, Juntendo University Hospital, Tokyo, Japan

**Keywords:** Chest trauma, Sauer's danger zone, Penetrating trauma, Chest computed tomography findings, Hemopneumothorax, Chest tube drainage

## Abstract

**Introduction and importance:**

Sauer's danger zone is an area on the anterior chest where trauma is considered to cause heart and macrovascular injury. Herein, we report the case of an injured patient showing evidently fatal findings on chest radiography and computed tomography (CT) presenting almost no actual fatal injuries on surgical examination.

**Case presentation:**

The patient was an 86-year-old man who was found by a family member with a 30-cm knife blade stuck in his left front chest (Sauer's danger zone). On chest CT findings, the knife was observed to be inserted through the 4th intercostal space, penetrating the lungs. The tip of the knife appeared to be anchored to the dorsal side of the 9th intercostal space.

**Clinical discussion:**

We found no damage to the heart, only a 2-cm-long and 1-cm-deep cut in the lingular segment area.

**Conclusion:**

We confirmed that the amount of bleeding in the inserted drain was an indicator of non-macrovascular injury. In cases of chest trauma, chest tube drainage and hemodynamics should always be observed, and the potential need for emergency surgery should be considered.

## Highlights

• The area where there is a risk of cardiovascular injury is called Sauer's danger zone.
• The amount of bleeding in the drain was an indicator of non-macrovascular injury.
• There is a discrepancy between chest CT and surgical findings.
• Chest tube drainage and hemodynamics should always be observed.
• The potential need for emergency surgery should be kept in mind.

## 1 Introduction

The area of precordial penetrating trauma associated with a high risk of cardiovascular injury is called Sauer's danger zone [1]. This is a rectangle surrounded by the suprasternal notch on the upper edge, the midline of the left clavicle on the left edge, the medial 1/3 of the right clavicle on the right edge, and the epigastric region and left and right sixth intercostal space on the lower edge. This site is associated with a high probability of heart and macrovascular injury; however, some cases of noninjury have been reported.

Herein, we report a case in which the patient only had a 2-cm-long and 1-cm-deep lung injury, despite having a knife penetration from Sauer's danger zone to the chest wall of the back.

This work has been reported in line with the SCARE criteria [2].

## 2 Presentation of case

**Fig. 1:**
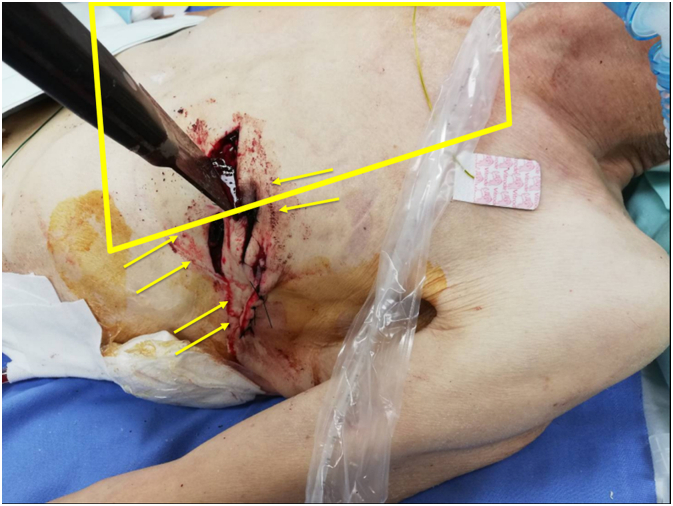
A knife was stuck in Sauer's danger zone on the patient’s chest, and three other cuts were found. The yellow square indicates Sauer’s danger zone. The yellow arrows indicate three cuts in the intrathoracic mediastinal direction. (For interpretation of the references to colour in this figure legend, the reader is referred to the web version of this article.)

An 86-year-old man with a history of dementia and type 2 diabetes was found by his son in an unconscious state with a 30-cm-blade knife stuck in his left chest. He was brought to our hospital with four puncture wounds in his left anterior chest and lateral chest. With a diagnosis of traumatic blood pneumothorax, he was referred to our department (Fig. 1).

**Fig. 2:**
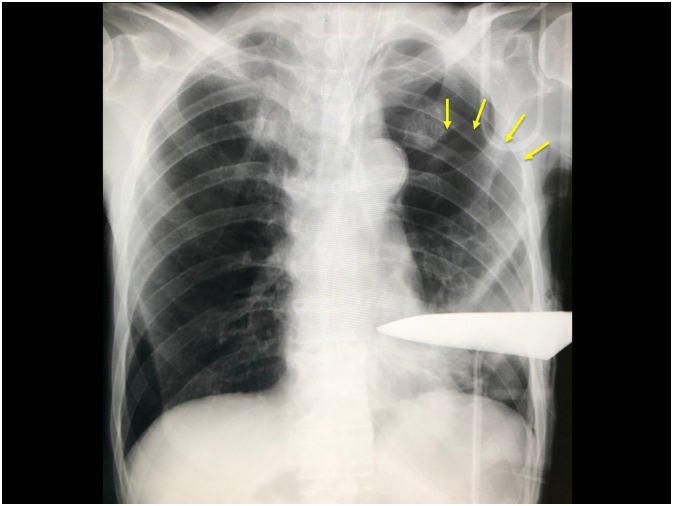
Chest radiography showing a knife penetrating the left chest wall and pneumothorax. The yellow arrows indicate the collapsed left lung. (For interpretation of the references to colour in this figure legend, the reader is referred to the web version of this article.)

**Fig. 3:**
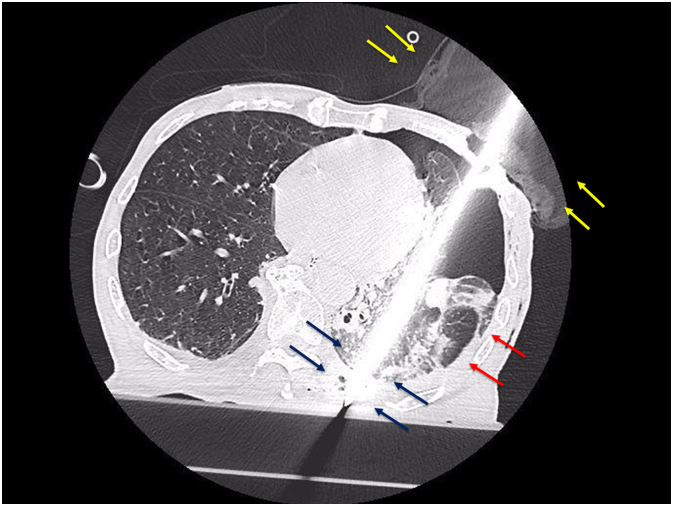
Chest CT showing penetration of the lower left lobe that reached the left dorsal chest wall. The yellow arrows show the knife secured with a towel or tape. The black arrows show the knife penetrating to the dorsal muscle layer of the 9th intercostal space. The red arrows show that there is no massive pleural effusion. (For interpretation of the references to colour in this figure legend, the reader is referred to the web version of this article.)

His vital signs at admission were: blood pressure, 167/76 mmHg; heart rate, 77 beats/min; and body temperature, 36.8 °C. Chest radiography showed a collapsed left lung with a knife stuck in the left thoracic cavity (Fig. 2). Chest computed tomography (CT) showed that the knife had been inserted from the ventral side of the left 4th intercostal space, first penetrating the lower left lobe, and then the dorsal side of the left 9th intercostal space, where it appeared to be fixed. In addition, the left lung was collapsed and left pleural effusion was observed (Fig. 3). CT revealed a small amount of pleural effusion and pneumothorax in the left thoracic cavity. According to the chest CT findings, the knife may have penetrated the left lung parenchyma. There were no abnormal findings in the abdominal cavity.

We inserted an intubation tube to improve his respiratory status and a chest tube to treat his left hemopneumothorax. At the time of insertion, 80 ml of bloody pleural effusion was drained, and 60 ml/h of continuous bloody pleural effusion was observed. The degree of air leakage was small. Blood sampling at the time of admission revealed anemia with an Hb level of 10.8 g/dl and electrolyte abnormalities (Na, 116 mmol/l; K, 2.4 mmol/l; and Cl, 71 mmol/l).

Since the amount of bleeding from the drain was less than expected and given the patient's age, surgery was not preferred initially. However, to (1) improve electrolyte abnormalities, (2) improve anemia, and (3) secure sufficient blood transfusion, we decided to perform elective surgery a day after the injury. We suspected the presence of electrolyte imbalances due to inadequate dietary intake, according to the family testimony. We considered the use of general anesthesia and attempted to improve the electrolyte imbalance before the surgery. The total drainage volume from the drain until the surgery was 860 ml. We also transfused MAP6U, FFP4U, and PC10U before the surgery. His Hb levels recovered to 12.5 g/dl, just before the surgery and no abnormalities in coagulation were observed. We expected the amount of bleeding before the surgery to be approximately 500 ml per day.

**Fig. 4:**
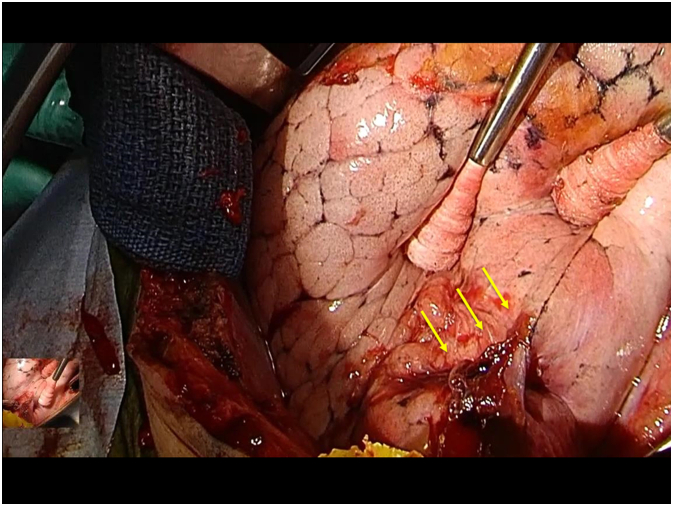
The surgical finding was only a 2-cm cut in the left lingular segment lung. The left lung is swollen with positive pressure ventilation during the surgery. The yellow arrows indicate a knife cut. (For interpretation of the references to colour in this figure legend, the reader is referred to the web version of this article.)

A 25-cm skin incision was made from the line of the posterior lateral incision of the 4th intercostal space toward the part where the knife was stuck. Considering the possibility of major bleeding occurring while pulling out the knife, we used vascular forceps to pull out the knife. However, we found only a 2-cm-long and 1-cm-deep puncture wound in the lingula segment area outside the perforation wound line, and no other obvious bleeding from the lung surface (Fig. 4). We attributed the source of bleeding to a laceration in the left lingular segment area and a puncture wound from the chest wall. The thoracic cavity was washed with 5000 ml of physiological saline, and the chest was closed as usual. The surgery time was 134 min, and the amount of bleeding was 130 ml. Intraoperative blood transfusions were not performed. The patient's postoperative course was uneventful. We did not require blood transfusions in this patient, even after surgery. The patient was ventilated until postoperative day (POD) 3. We removed the ventral and dorsal drains on POD 5 and POD 7, respectively. However, he developed pneumothorax again, so we inserted a chest drain on POD 10 and removed it on POD 17. As his activities of daily living declined, he was transferred to a rehabilitation hospital on POD 25.

## 3 Discussion

It is rare for a puncture wound in Sauer's danger zone to avoid serious heart or lung damage [1], [3].

The patient may have escaped a fatal lung injury even though the knife penetrated the lungs, as observed on the CT findings. A partial cut was made in the lingula segment of the lung when the patient was stabbed in the precordium thrice with a knife, resulting in a traumatic pneumothorax and lung collapse. The patient, then lost consciousness and fell with the knife stuck in his chest, creating a cut that penetrated his fourth dorsal chest wall. Therefore, we believed that the only lung injury was a small cut in the lingular segment of the lung.

The timing and choice of the emergency surgery for this case may be open to debate. Although the advantage of early surgery is that the bleeding site can be controlled immediately, the disadvantage is that blood transfusions cannot be sufficiently prepared at the time of surgery. In contrast, the advantage of late surgery is that blood transfusions can be sufficiently prepared, and the disadvantages are 1) the movement of the penetrating knife, 2) the possibility of infections of the thoracic cavity if the knife is dirty, and 3) the bleeding would not be controlled early on, if the drainage was unsuccessful. Keeping these in mind, we took the following measures: 1) the knife was taped to prevent any movement prior to the surgery; 2) the chest cavity was washed with 5000 cm^3^ saline during the surgery; 3) chest radiography was performed as needed, and the inside of the chest cavity was observed.

Simens et al. reported the following five surgical indications for penetrating chest trauma [4]: 1) a wound in the upper mediastinum, 2) systolic blood pressure at the time of delivery <90 mmHg, 3) bleeding of ≥800 ml when a chest drain is inserted, 4) persistent hemothorax on chest X-ray, and 5) a merger of cardiac tamponade. In this case, only the first one was applicable. Demetriade et al. reported that patients in shock but not in urgency should receive thoracotomy in the operating room after considering treatment priorities [5]. 3% to 30% patients undergoing thoracotomy for hemorrhage have been shown to require lung resection for control of injuries [6], [7]. In addition, since the mortality rate is extremely high in cases where surgery is performed for lung injury, sufficient blood transfusion preparation and surgical instruments may be required [8], [9], [10], [11]. As a limitation of the approach in this case, we overlooked the possibility of major bleeding from the chest cavity at any time. However, owing to the improvement in the patient's general condition, the minor bleeding from the drain, availability of blood transfusion, the surgery was performed a day after the injury. In cases of chest trauma, chest tube drainage and hemodynamics should always be observed, and the possibility of an emergency surgery should be considered.

## 4 Conclusion

In conclusion, we encountered a rare case of penetrating chest trauma in Sauer's danger zone without severe heart or lung damage. In this case, information on the amount of bleeding from the chest tube was more useful than a chest CT scan in determining the condition of the chest cavity. Our study indicates that surgical treatment for chest trauma should be performed as soon as the patient's general condition improves, the operating room is prepared and blood transfusion is possible.

## Sources of funding

There were no sponsors of support for this study.

## Ethical approval

Ethical Clearance was obtained from the Institutional Research and Ethics Review Committee (IRB) of Juntendo University Hospital for the publication of the case report.

## Consent

Written informed consent was obtained from the patient for publication of this case report and accompanying images. A copy of the written consent is available for review by the Editor-in-Chief of this journal on request.

## Authors' contributions

HI, YK, and SU drafted the manuscript. HI and YK wrote the paper, including the first draft, and KS decided to publish the paper. HI, YK, and SU performed the surgery. HI, YK, and SU followed-up after surgery. All authors have read and approved the final manuscript.

## Research registration

Not applicable.

## Guarantor

Hideomi Ichinokawa.

## Provenance and peer review

Not commissioned, externally peer reviewed.

## Declaration of competing interest

All authors declare having no conflicts of interest for this article.
